# Disturbances of the Stress Response: The Role of the HPA Axis During Alcohol Withdrawal and Abstinence

**Published:** 1998

**Authors:** Bryon Adinoff, Ali Iranmanesh, Johannes Veldhuis, Lisa Fisher

**Affiliations:** Bryon Adinoff, M.D., is the Distinguished Professor of Alcohol and Drug Abuse Research and associate professor in the Department of Psychiatry, University of Texas Southwestern Medical Center at Dallas, and medical director of the Substance Abuse Team at the Dallas Veterans Affairs Medical Center, VA North Texas Health Care System, Dallas, Texas. Ali Iranmanesh, M.D., is associate professor of endocrinology in the Department of Medicine, University of Virginia School of Medicine, Charlottesville, Virginia, and is chief of the Endocrine Section and director of the Endocrine Laboratory, Salem VA Medical Center, Salem, Virginia. Johannes Veldhuis, M.D., is a professor of endocrinology in the Department of Medicine and director of the General Clinical Research Center, University of Virginia School of Medicine, Charlottesville, Virginia. Lisa Fisher, Ph.D., is an assistant professor in the Department of Psychiatry, University of Texas Southwestern Medical Center, and a staff psychologist at the Dallas Veterans Affairs Medical Center, VA North Texas Health Care System, Dallas, Texas

**Keywords:** AOD withdrawal syndrome, physiological stress, hypothalamic-pituitary axis, pituitary-adrenal axis, cortisol, AOD abstinence, chronic AODE (alcohol and other drug effects), corticotropin RH, arginine, vasopressin, adrenocorticotropic hormone, secretion, metabolic disorder, AODR (alcohol and other drug related) disorder, mood and affect disturbance, personality disorder, infection, drug therapy, literature review

## Abstract

Interactions among the brain, the pituitary gland, and the adrenal glands (i.e., the hypothalamic-pituitary-adrenal [HPA] axis) help regulate the body’s response to stress. The adrenal hormone cortisol plays a key role in stress reduction through its effects on multiple body systems. Excessive cortisol activity during both chronic alcohol administration and withdrawal may underlie some of the clinical complications of alcoholism, including increased risk of infectious diseases; bone, muscle, and reproductive system changes; altered energy metabolism; and disorders of mood and intellect. Despite excessive cortisol levels during intoxication and withdrawal, however, the HPA axis becomes less responsive to stress during abstinence, potentially resulting in an impaired capacity to cope with relapse-inducing stressors.

Stress is a ubiquitous and unavoidable experience of daily life whether it arises from the external environment (e.g., a job interview or traffic accident) or from within the body (e.g., an infection or a panic attack). The body has powerful mechanisms to cope with stress. Among these mechanisms is a hormone called cortisol, which is produced and secreted by the adrenal glands, located atop the kidneys (see figure 1). Without cortisol a human or animal cannot respond appropriately to different types of physical or mental stress. However, because cortisol affects a wide range of critical physiological processes, the activity of cortisol must be tightly controlled by the body.

Cortisol secretion is regulated by interactions among three structures: the hypothalamus, the pituitary gland, and the outer layer (i.e., cortex) of each adrenal gland (see figure 1). These three structures, collectively known as the hypothalamic-pituitary-adrenal (HPA) axis, provide a regulatory network linking the brain with the body’s behavioral and physiological responses to stress.

Alcohol consumption disrupts this regulatory balance. Excessive cortisol secretion occurs during both chronic alcohol consumption and alcohol withdrawal. This heightened secretion rate may alter energy metabolism, mental status, the structural integrity of bone and muscle tissue, and the body’s ability to resist infection. In addition, abstinent alcoholics exhibit a diminished ability of the HPA axis to respond to stress, potentially impairing the body’s capacity to cope with stressors that might induce relapse to drinking. This article discusses the organization and regulation of the HPA axis, disorders associated with impaired HPA functioning, and the combined effects of long-term alcohol consumption and HPA disturbance on health.

## The HPA Axis and Stress

### Organization and Regulation of the HPA Axis

**Figure 1:**
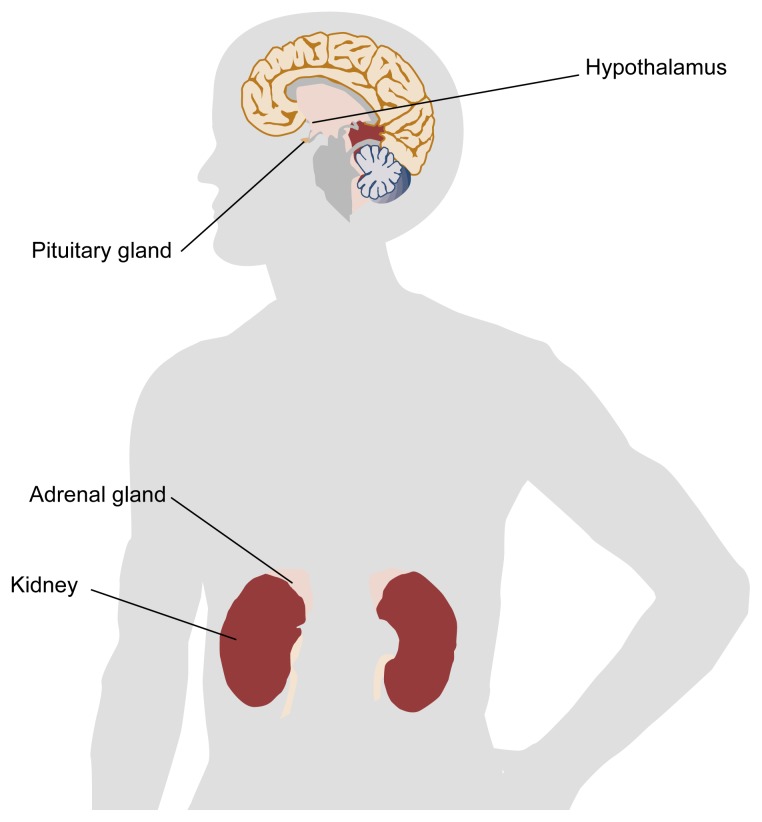
Location of the components of the hypothalamic-pituitary-adrenal (HPA) axis. The hypothalamus is located in the brain, directly above the pituitary gland. The adrenal glands are located in the lower back, one atop each kidney.

In response to stimulation from the brain, the hypothalamus produces corticotropin releasing hormone (CRH) and/or arginine vasopressin (AVP)^1^ (Rivier 1996). CRH and AVP are then channeled directly to the pituitary gland situated just beneath the hypothalamus (see figure 1). Within the pituitary, these two hormones act together to stimulate the release of adrenocorticotropic hormone (ACTH). ACTH arrives at the adrenal cortex via the bloodstream, where it stimulates the secretion of cortisol. Cortisol then travels through the bloodstream, exerting effects on multiple organs and tissues.

The HPA axis demonstrates a consistent, daily (i.e., circadian) pattern. In humans, cortisol levels decrease during the late evening hours, reaching their lowest point during the early morning hours. Cortisol secretion begins to increase several hours prior to awakening, and peak levels occur in the late morning hours. In addition, complex short-term fluctuations of cortisol levels occur within the day (Gudmundsson and Carnes 1997; Johnson and Veldhuis 1995).

The HPA axis incorporates a system of controls that dampen its own activation (i.e., a negative feedback system). The hypothalamus and the pituitary gland are sensitive to inhibition by cortisol. Thus, when activation of the stress response produces increases in CRH and ACTH, the resultant elevation in cortisol (after a time delay) suppresses further CRH and ACTH production.

These interactions help ensure that the body’s stress response system does not overreact in its response to a stressor (Munck et al. 1984). This constant adjustment and readjustment of hormone levels around a target concentration has been called the “allostatic load.” Humans or animals with a high allostatic load are in a state of perpetual anxious anticipation. The chronic elevation in cortisol induced by a persistent or repetitive allostatic load can activate the synthesis of CRH by the brain, thereby increasing levels of anxiety and fear (McEwen 1994; Schulkin et al. 1994).

## Disorders Associated with Disrupted HPA Axis Functioning

A disordered HPA axis response can cause major physiological damage. When excess cortisol is produced, often because of an ACTH-secreting tumor in the pituitary gland (i.e., Cushing’s syndrome), patients may experience symptoms related to excess levels of the normal physiological activity of cortisol (see textbox). These symptoms include increased blood sugar, bone weakness, decreased resistance to infection, increased fat metabolism, high blood pressure, major alterations in mood, impaired mental functioning, and disturbed sleep.

**Physiological Effects of Cortisol**

Although the physiological effects of cortisol are well documented, the role of these effects in stress resistance is uncertain. Cortisol is one of several steroid hormones produced by the outer shell (i.e., cortex) of the adrenal glands (see figure 1). Classified as a glucocorticoid because of its effect on glucose metabolism, cortisol in simple terms can be said to counterbalance the metabolic effects of insulin. Insulin decreases glucose levels in the bloodstream, whereas cortisol increases glucose levels, breaking down fat and protein to supply raw materials for increased glucose synthesis. When cortisol is produced in excess, the breakdown of protein may lead to muscle weakness and decreased bone mass. Cortisol also suppresses a wide range of immune functions. This property forms the basis for the use of synthetic glucocorticoids (e.g., prednisone) to treat excess inflammation resulting from trauma or infection.

Excess cortisol also impairs the brain’s ability to metabolize energy, which can leave the brain vulnerable to low levels of oxygen (such as following a stroke or heart attack) or low blood sugar (i.e., hypoglycemia). Long-term damage to the hippocampus—a brain structure vital to learning, memory, and the regulation of HPA axis function—appears to occur in chronic medical and psychiatric illnesses associated with persistently elevated levels of cortisol (Sapolsky 1996).

Conversely, a deficiency of cortisol can lead to low blood glucose levels and a decreased ability to convert lipid and protein molecules to glucose. Symptoms of cortisol deficiency include low-grade fever, easy fatigability, weakness, weight loss, muscle aches, abdominal pain, vomiting, low blood pressure, and personality changes such as irritability and restlessness. More severe and life-threatening consequences, such as cardiovascular shock, can occur when cortisol-deficient patients are exposed to an episode of severe stress (e.g., surgery) without prior administration of cortisol or another glucocorticoid (see textbox).^2^

Alterations in HPA axis functioning are associated with a number of psychiatric disorders and personality characteristics. Thus, both overactivity and underactivity of the HPA axis are associated with depressive mood states, aggression, and lack of behavioral control (Stansbury and Gunnar 1994; Yehuda et al. 1993). These somewhat disparate findings suggest that HPA axis disturbances can be associated with abnormal levels of nervous system excitability on *either* side of an optimal range (Chrousos 1992).

## Alcohol-Related Disturbances in the HPA Axis

### Alcohol Consumption and Withdrawal

Investigators have explored the time course and mechanisms of alcohol-induced HPA axis activation in rodents (see Rivier 1996; Eskay et al. 1995). Although alcohol itself does not appear to exert a powerful direct stimulatory effect on the adrenal cortex, increased rates of CRH synthesis, coupled with increased secretion of ACTH and cortisol, are generally observed in animals following long-term alcohol administration. The effects of short- and long-term alcohol consumption in humans are less clear, although most studies suggest that chronic administration of alcohol increases cortisol in humans.

Both human and animal studies clearly demonstrate an increase in HPA axis activation with elevated cortisol concentrations during alcohol withdrawal (Iranmanesh et al. 1989). In alcoholics with high daily alcohol consumption, cortisol levels return to normal after 7 days of abstinence (Adinoff et al. 1991).

### Inhibition of the HPA Axis in Abstinent Alcoholics

**Figure 2:**
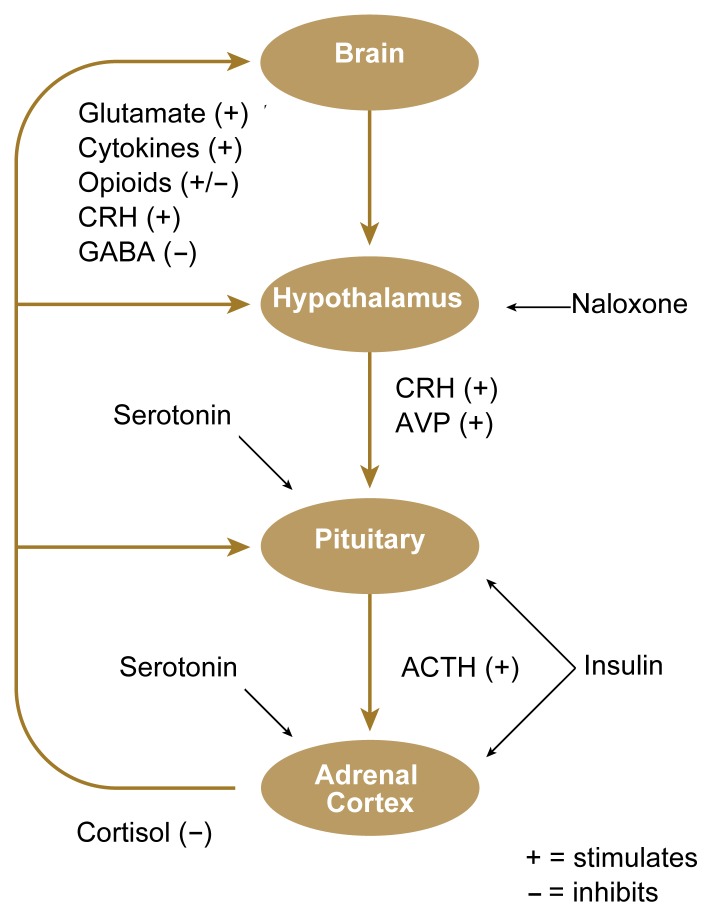
Regulation of the hypothalamic-pituitary-adrenal (HPA) axis. Almost any type of stress, including trauma, infection, intense heat or cold, or mental effort, is followed within minutes by increased secretion of cortisol. The occurrence of stress is communicated to the HPA axis by various chemical messengers. The brain monitors and integrates these communications and, when appropriate, activates secretion of corticotropin-releasing hormone (CRH) (and arginine vasopressin [AVP]) from the hypothalamus. These hormones stimulate the pituitary gland to secrete adrenocorticotropic hormone (ACTH), which stimulates the adrenal cortex to secrete cortisol. Substances that promote CRH secretion include (among others) stimulatory neurotransmitters (e.g., glutamate), mediators of inflammation (i.e., cytokines), and CRH itself. CRH secretion is decreased by inhibitory neurotransmitters (e.g., gamma-aminobutyric acid [GABA]); in addition, endogenous opioids provide continuous (i.e., tonic) background inhibition of CRH secretion. Of the many chemical messengers that influence secretion of ACTH (by the pituitary) and cortisol (by the adrenal cortex), only serotonin is shown because of its modulatory effect on multiple brain functions. In addition, cortisol modulates its own release (i.e., feedback inhibition), dampening potentially excessive HPA response. The right of the diagram lists selected substances that can be administered to evaluate the responsivity of the HPA axis at various levels. Naloxone suppresses inhibition of CRH secretion by opioids; insulin administration lowers blood sugar, activating ACTH and cortisol secretion (see textbox).

Studies have shown low CRH concentrations and low morning levels of plasma ACTH in abstinent alcoholics. Additional studies in abstinent alcoholics have revealed decreased hormonal responsiveness at each level of the HPA axis. For example, naloxone (see figure 2) induces a blunted CRH response by the hypothalamus in abstinent patients; secretion of ACTH by the pituitary is lessened following administration of CRH or insulin; and cortisol secretion is suppressed following exposure to alcohol, insulin, CRH, ACTH, and various environmental and mental stressors (for a review, see Costa et al. 1996).

Thus, alcohol dependence appears to be associated with two distinct patterns of HPA axis disturbances. Marked HPA axis activation is present during chronic alcohol consumption and during alcohol withdrawal, whereas a suppression of HPA axis functioning, particularly in response to various stressors, is observed during abstinence. Accounting for these two disparate physiological mechanisms is difficult, because decreased responsiveness of the HPA axis is evident at each separate level of the system (i.e., the hypothalamus, the pituitary, and the adrenals each show evidence of blunted responsiveness in abstinent alcohol-dependent patients). These findings may be the result of genetic influences coupled with multiple physiological attempts to compensate for persistent alcohol-and withdrawal-induced HPA axis activation.

## Implications of HPA Axis Disturbances in Alcoholism

### Alcohol-Related Medical Effects

Daily heavy alcohol consumption may induce two putative secretion patterns in the HPA axis. First, high levels of cortisol may alternate with normal levels as the patient drinks progressively more alcohol during the day (producing high cortisol levels), stops drinking upon the initiation of sleep (producing normalized levels of cortisol), begins to experience the early stages of withdrawal upon awakening (producing high cortisol levels), and then re-initiates the drinking cycle (initially lowering cortisol concentrations as withdrawal subsides). This back-and-forth, “seesaw” activity of the HPA axis and other stress-response systems would be expected to produce a persistent state of high allostatic load. Alternately, cortisol levels may remain persistently high throughout the drinking-withdrawal cycle, which may be maintained for several months or years.

Either of the aforementioned patterns would be expected to produce disturbances associated with high concentrations of cortisol. One such condition is pseudo-Cushing’s syndrome, in which alcoholics develop biochemical and clinical characteristics of Cushing’s syndrome (Veldman and Meinders 1996). Additionally, long-term intermittent or continuous states of high cortisol associated with alcoholism could contribute to the development or increase the severity of loss of bone mass (i.e., osteoporosis), diabetes mellitus, muscle wasting, high blood pressure, decreased immune function, low levels of the male reproductive hormone testosterone, and liver damage.

Persistently elevated levels of cortisol, in combination with certain effects of withdrawal, low blood sugar, or deficiency of the B vitamin thiamine, may produce significant neurotoxicity resulting in hippocampal damage (Adinoff 1994). Such damage may result in alcohol-related symptoms such as personality changes, memory loss, and depression. High levels of CRH and cortisol during withdrawal also could contribute to the severity of the withdrawal syndrome itself (Menzaghi et al. 1994; Roberts et al. 1995). In rats, blocking withdrawal-associated increases in CRH decreases alcohol withdrawal symptoms. When cortisol is increased following the cessation of alcohol administration, withdrawal symptoms worsen, and when the effects of cortisol are blocked during alcohol withdrawal, withdrawal symptoms decrease. In addition, increased levels of CRH and cortisol during withdrawal may potentiate each other’s excitatory effects, because cortisol increases CRH’s ability to induce seizures.

Finally, increased levels of cortisol may themselves be reinforcing, acting on the brain to perpetuate behaviors (e.g., alcohol consumption) that maintain high cortisol levels. Evidence for this hypothesis has been obtained from both humans and animals. Cortisol is reinforcing in rats, modulates the self-administration of alcohol in animals, and increases brain sensitivity to other addictive drugs (e.g., stimulants and opioids) (Deroche et al. 1995). Anecdotal reports have noted the self-administration of cortisol in humans in a manner consistent with an addictive disorder.

### Mood and Personality During Abstinence

During abstinence, the apparent decreased responsivity of the hypothalamus, pituitary gland, and adrenal cortex may have significant effects on behavior and mood. Two dimensions of personality styles frequently observed in alcoholics are (1) emotional instability (i.e., neuroticism) and (2) lack of restraint (i.e., behavioral disinhibition) (Sher and Trull 1994). High levels of anxiety and mood disturbances also are commonly reported in alcoholic patients. The presence of anxiety and mood disturbances as well as high emotional instability and behavioral disinhibition are predictive of relapse in abstinent alcoholics (Fisher et al. in press). As noted previously, decreased cortisol concentrations and muted HPA axis reactivity are associated with chronic stress disorders, impulsiveness, and behavioral disinhibition. The presence of persistently suppressed HPA axis responsiveness in these patients may therefore be related to affective states and behaviors highly associated with future drinking.

## Implications for Treatment

Researchers are developing medications to treat various aspects of alcoholism. The role of alcohol in modulating the stress response suggests additional approaches for pharmacotherapy. One example might be to modify the function of endogenous opioids (i.e., chemical messengers in the brain that have biological effects similar to those of opiate drugs, such as heroin). Among other functions, endogenous opioids provide continuous (i.e., tonic) background inhibition of CRH secretion (see figure 2). Naltrexone, a long-acting antagonist of opioid function, is extremely effective for treating alcohol dependence. The clinical effectiveness of naltrexone may in part reflect its ability to heighten hypothalamic responsiveness in alcohol-dependent patients. Such pharmacological “resetting” of the triggerpoint required to provoke cortisol release in the presence of uncertainty or conflict may facilitate more appropriate modulation of the stress-response system in abstinent patients, thus prolonging abstinence.

## Footnotes

1 Vasopressin is a hormone with several functions in the body, including regulating water balance.

2 The glucocorticoid corticosterone, also secreted by the adrenal cortex, is of minor importance in humans compared with cortisol.
